# In vitro biomechanical comparison after fixed- and mobile-core artificial cervical disc replacement versus fusion

**DOI:** 10.1097/MD.0000000000008291

**Published:** 2017-10-13

**Authors:** Jigang Lou, Yuanchao Li, Beiyu Wang, Yang Meng, Tingkui Wu, Hao Liu

**Affiliations:** a Department of Orthopedics, West China Hospital, Sichuan University, Chengdu, Sichuan; b Department of Biomechanical Research Laboratory, Shanghai Jiao Tong University, Shanghai, China.

**Keywords:** biomechanics, cervical disc replacement, disc prosthesis, fixed core, mobile core

## Abstract

In vitro biomechanical analysis after cervical disc replacement (CDR) with a novel artificial disc prosthesis (mobile core) was conducted and compared with the intact model, simulated fusion, and CDR with a fixed-core prosthesis. The purpose of this experimental study was to analyze the biomechanical changes after CDR with a novel prosthesis and the differences between fixed- and mobile-core prostheses.

Six human cadaveric C2–C7 specimens were biomechanically tested sequentially in 4 different spinal models: intact specimens, simulated fusion, CDR with a fixed-core prosthesis (Discover, DePuy), and CDR with a mobile-core prosthesis (Pretic-I, Trauson). Moments up to 2 Nm with a 75 N follower load were applied in flexion–extension, left and right lateral bending, and left and right axial rotation. The total range of motion (ROM), segmental ROM, and adjacent intradiscal pressure (IDP) were calculated and analyzed in 4 different spinal models, as well as the differences between 2 disc prostheses.

Compared with the intact specimens, the total ROM, segmental ROM, and IDP at the adjacent segments showed no significant difference after arthroplasty. Moreover, CDR with a mobile-core prosthesis presented a little higher values of target segment (C5/6) and total ROM than CDR with a fixed-core prosthesis (*P* > .05). Besides, the difference in IDP at C4/5 after CDR with 2 prostheses was without statistical significance in all the directions of motion. However, the IDP at C6/7 after CDR with a mobile-core prosthesis was lower than CDR with a fixed-core prosthesis in flexion, extension, and lateral bending, with significant difference (*P* < .05), but not under axial rotation.

CDR with a novel prosthesis was effective to maintain the ROM at the target segment and did not affect the ROM and IDP at the adjacent segments. Moreover, CDR with a mobile-core prosthesis presented a little higher values of target segment and total ROM, but lower IDP at the inferior adjacent segment than CDR with a fixed-core prosthesis.

## 1 Introduction

As an alternative to traditional anterior cervical discectomy and fusion, cervical disc replacement (CDR) has become increasingly popular with spine surgeons to treat radiculopathy or myelopathy (mainly soft disc herniation) in recent years, for allowing the preservation of the mobility at the surgical level and reducing the stress transferred to the adjacent levels.^[1–3]^ An artificial cervical disc is designed to replace degenerative disc and restore physiologic segmental function. To date, a number of commercial and experimental artificial cervical disc prostheses are available on the market or under clinical trials. However, most of them present a flat surface instead of an arcuate surface.^[4]^ Considering that the morphology of inferior endplates of the cervical spine was mainly concave,^[5,6]^ we designed and manufactured a novel artificial disc prosthesis (Pretic-I) based on the physiological curvature of cervical endplate. This novel disc prosthesis was designed in attempt to increase the contact area between the prosthesis endplate and vertebral endplate, and to dissipate the axial load further.

In addition, almost all disc prostheses currently available employ a spherical interface with or without incorporated translation.^[7]^ The novel prosthesis is no exception, with translation design features which allow for a mobile center of rotation (COR) and have the theoretical advantage of providing normal kinematics over a series of device positions.^[8]^ However, the traditional Discover (DePuy Spine, Raynham, MA) cervical disc, without translation design features, provides a fixed COR, thus, requiring precise device placement to replicate anatomic centers of rotation in order to restore normal kinematics. Hence, it is very necessary to get a comprehensive understanding of the influence of different prosthesis designs of CDRs on the biomechanics of the cervical spine.

Cadaveric specimens from the human spine are often used as a biomechanical model. Through an in vitro biomechanical study, the primary objective was to observe biomechanical changes like cervical range of motion (ROM) and intradiscal pressure (IDP) after CDR with a novel prosthesis, in comparison with those in the intact model, simulated fusion and CDR with a fixed-core prosthesis. A secondary objective was to analyze the biomechanical differences between fixed- and mobile-core prostheses.

## 2 Materials and methods

The present study was approved by the ethics committee of the West China Hospital, Sichuan University.

### 2.1 Specimens preparation

Six fresh-frozen human cadaveric C2–C7 specimens that came from donors were used for biomechanical testing (5 males, 1 female; age range: 47–68 years; mean: 58 years). Plain radiographs were taken to rule out any specimens with obvious flaws, such as fractures, deformities, tumors, metastatic disease, or severe disc degeneration (osteophytes, severe disc space narrowing, or facet hypertrophy). Each specimen was then kept frozen in triple sealed bags at −20°C. In preparation, all specimens were thawed to room temperature and dissected clear of all the surrounding soft tissue and muscle, while preserving all ligamentous structures and their attachments, as well as the intervertebral discs and facet joint capsules. Specimens were prepared for biomechanical testing in a spine tester. The proximal (C2) and distal (C7) ends of the specimen were embedded in polymethylmethacrylate cement in cylindrical aluminum fixtures. C7 was prepared for additional stabilization by partially inserting 3 perpendicular screws into the exposed endplates. In addition, in order to avoid tissue dehydration, all specimens were kept moistened by spraying saline (0.9% NaCl) during the tests.

### 2.2 Biomechanical testing apparatus

**Figure 1:**
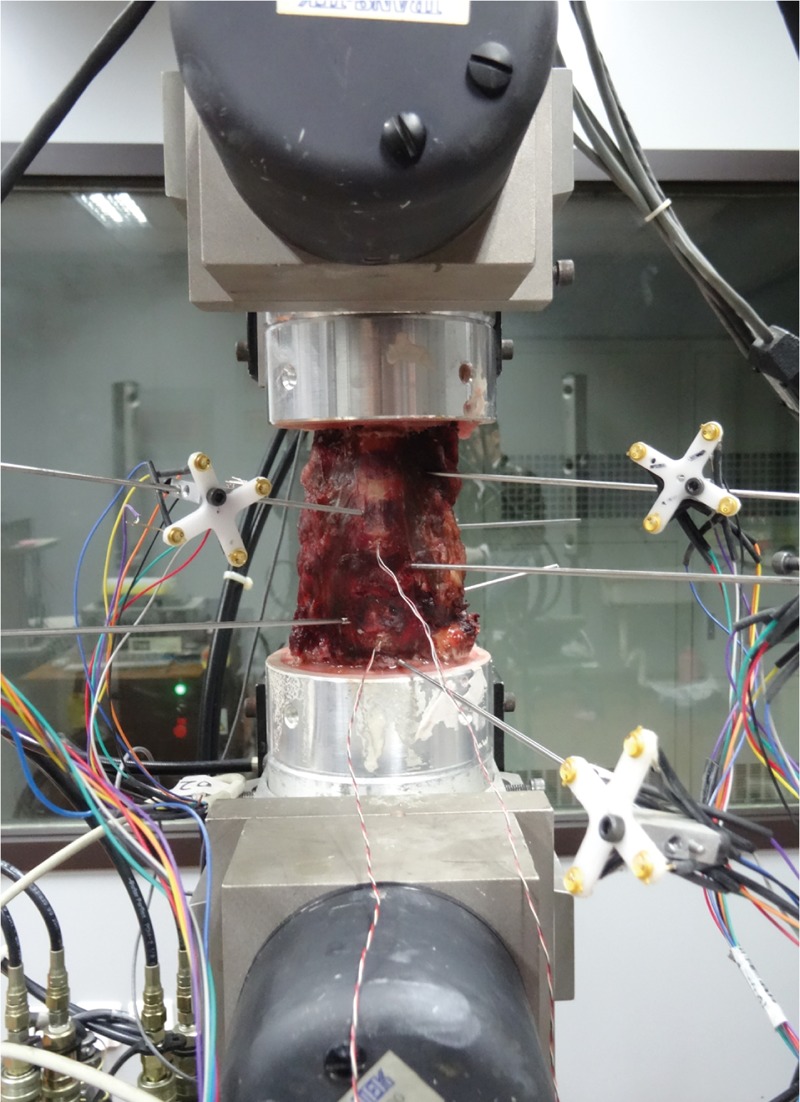
Anterior view of an intact specimen. Each Kirschner pin connected 4 optical markers to the vertebral bodies of the specimen. The miniature pressure sensors Model 060 were inserted into the discs C4/5 and C6/7.

Biomechanical testing was performed on multidegree of freedom servo-hydraulic testing system (MTS 858 bionix machine, MTS Systems Inc, Minneapolis, MU). Loading was applied to the proximal end (C2) of the specimen, whereas the distal portion (C7) remained fixed to the socket of the apparatus. Pure moments of 2.0 Nm maximum with a constant rate of 0.2 Nm/s were applied sequentially about the 3 primary anatomical axes to induce flexion, extension, left and right lateral bending, and left and right axial rotation using a load control protocol.^[9]^ A 75 N follower load was applied to simulate physiologic compressive loads.^[10]^ Optical markers were connected by Kirschner pins to each vertebral body of the specimen. The Optotrak 3020 (Northern Digital, Waterloo, ON, Canada) was used to record and analyze the 3-dimensional motion of the specimens. In addition, the miniature pressure sensors Model 060 (Precision Measurement Company, Ann Arbor, MI) were placed using a guide tube in the nucleus at the center of the discs C4/5 and C6/7 to measure the IDP (Fig. 1).^[11]^ While each load was applied, the voltage outputs from the pressure sensors were recorded continuously.

### 2.3 Device design

**Figure 2:**
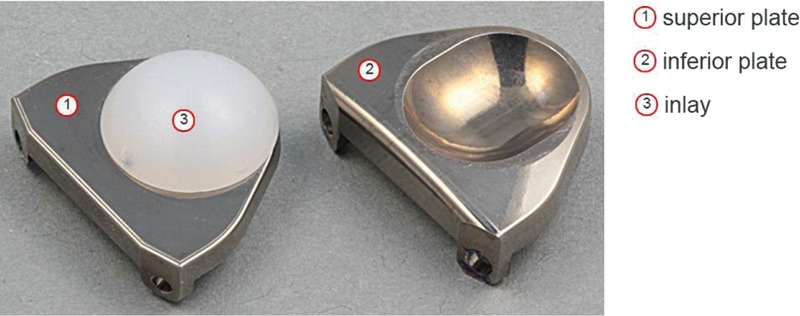
A novel cervical disc prosthesis (Pretic-I). It is composed of a superior plate, an inferior plate, and an inlay.

The novel cervical disc prosthesis (Pretic-I, Trauson) was designed and manufactured at the West China Hospital, Sichuan University. It consisted of 2 endplates (Ti6A14V) and an ultra-high-molecular-weight polyethylene (UHMWPE) core (Fig. 2), making its biocompatibility and wear-resistance much better.^[12]^ The UHMWPE-bearing surface attached to the superior endplate permitted itself to move back and forth along a slot in the horizontal direction. Thus, the novel prosthesis incorporated a ball-in-trough design and provided a mobile COR. The back surface of each endplate has 2 rows of dentate crests to improve the initial stability of the prosthesis, and is sprayed with a hydroxyapatite coating to allow bone in-growth to the implant.

### 2.4 Surgical procedure

**Figure 3:**
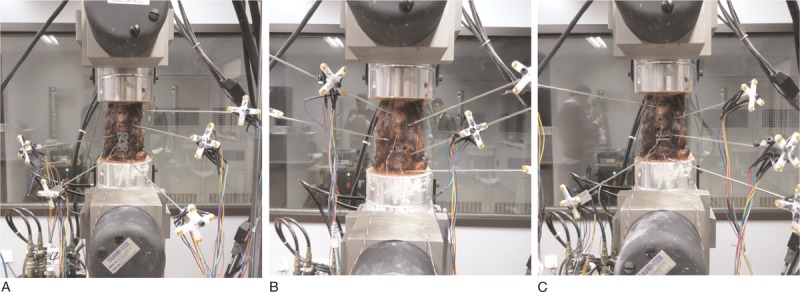
Anterior view of the specimens in the different models: simulated fusion with a locking anterior plate system (A), CDR with Type D prosthesis (B), and CDR with Type P prosthesis (C). CDR = cervical disc replacement.

**Figure 4:**
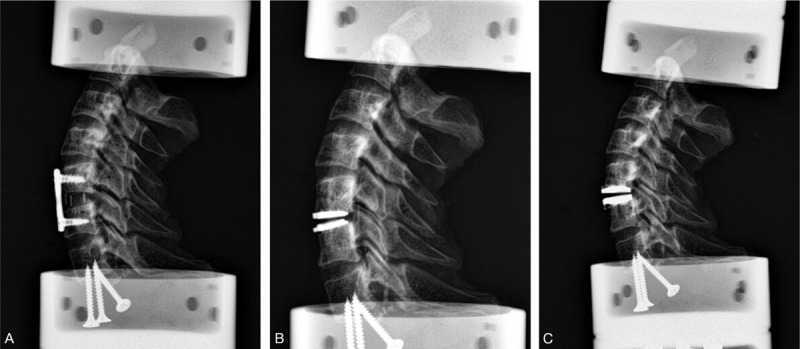
Radiographs of 3 different specimens. The correct position of the locking anterior plate (A), the Type D prosthesis (B), and the Type P prosthesis (C).

Six specimens were tested sequentially in 4 different spinal models: intact specimens, simulated fusion, CDR with the Discover prosthesis (CDR Type D), and CDR with the Pretic-I prosthesis (CDR Type P). After analysis of intact specimens, a complete disc discectomy of C5/6 was performed in all specimens. The posterior longitudinal ligament was routinely resected. Then a cervical plate system was supplemented to simulate a single-level fusion (Fig. 3A). After this testing, the plate, the screws, and the cage were removed. Thereafter, the endplates were flattened using a curette and a high-speed burr. CDR was performed according to the manufacturer's recommended tools and procedures. Using a guide tool, reference pins were inserted into the vertebral bodies above and below the target segment, leaving holes in the vertebral bodies. Trial sizes were used to assess the appropriate size of the prosthesis. The optimal prosthesis was then attached as a single unit to an insertion tool and driven into place with a hammer. The Discover prosthesis (fixed core) was inserted at the C5/6 segment (Fig. 3B). After this testing, the Discover prosthesis was removed, and then the Pretic-I prosthesis (mobile core) was implanted also at the C5/6 segment (Fig. 3C). Accounting for viscoelastic creep, 2 precycles were applied to the specimens and the 3rd cycle was used for data analysis. Radiographs were taken to confirm the correct position of all implants (Fig. 4A–C).

### 2.5 Statistical analysis

Mean values and standard deviations were determined for each parameter. SPSS software (Version 19.0, SPSS Inc, Chicago, IL) was used for statistical analysis. The parameters included the total ROM, segmental ROM, and IDP at the adjacent segments. All data among the 4 different spinal models (intact specimens, simulated fusion, CDR Type D, and CDR Type P) were analyzed using 1-way analysis of variance and Bonferroni post hoc to determine whether or not outcome measures were significantly different. A value of *P* < .05 was considered statistically significant.

## 3 Results

### 3.1 Total ROM and segmental ROM

**Figure 5:**
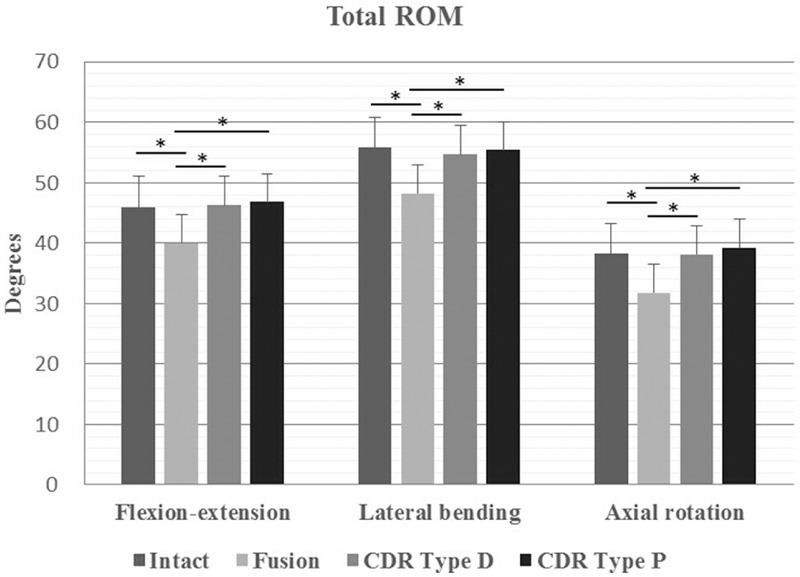
Total ROM. Total ROM in flexion–extension, lateral bending, and axial rotation in 4 different models: intact, fusion, CDR Type D, and CDR Type P. Statistically significant differences are denoted by ∗, with bars connecting the corresponding columns. CDR = cervical disc replacement, ROM = range of motion.

The mean total ROM in flexion–extension, lateral bending, and axial rotation was always recorded at the maximum loading of ±2 Nm. The differences in total ROM among the 3 models (intact specimens, CDR Type D, and CDR Type P) were not statistically significant in all 3 directions of motion with the following values: for flexion–extension 45.93°±3.43° in the intact model, 46.32°±2.68° in the CDR Type D model, and 46.86°±2.76° in the CDR Type P model; for lateral bending 55.80°±3.91° in the intact model, 54.69°±5.20° in the CDR Type D model, and 55.44°±3.91° in the CDR Type P model; for axial rotation 38.24°±4.96° in the intact model, 38.12°±4.42° in the CDR Type D model, and 39.25°±3.72° in the CDR Type P model. However, the total ROM after fusion was all significant lower than that in the other 3 models in all 3 directions of motion (*P* < .05) (Fig. 5).

**Figure 6:**
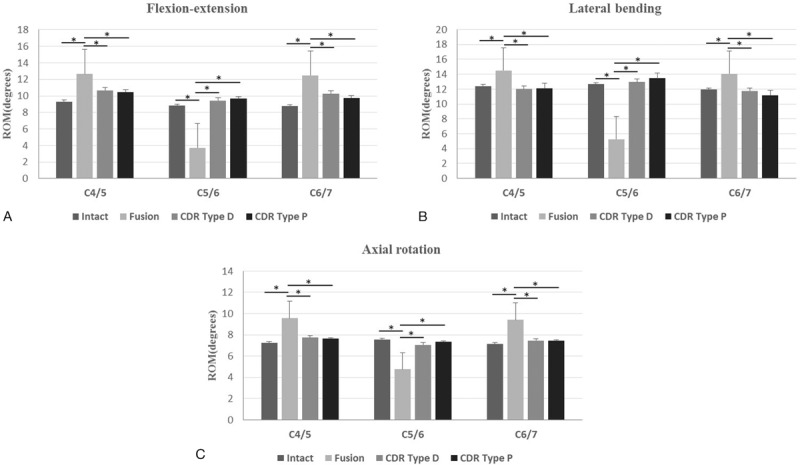
Segmental ROM. Segmental ROM in 4 different models (intact, fusion, CDR Type D, and CDR Type P) in flexion–extension (A), lateral bending (B), and axial rotation (C). Statistically significant differences are denoted by ∗, with bars connecting the corresponding columns. CDR = cervical disc replacement, ROM = range of motion.

The mean values of segmental ROM at C4/5, C5/6, and C6/7 in all 3 directions of motion in each of the 4 models are shown in Table 1. Similar to the total ROM, the differences in segmental ROM among the 3 models (intact specimens, CDR Type D, and CDR Type P) were also not statistically significant in all 3 directions of motion. However, the segmental ROM at C5/6 after fusion was significant lower than that in the other 3 models in all 3 directions of motion (*P* < .05). Meanwhile, the ROM at the adjacent segments after fusion was significant larger than that in the other 3 models in all 3 directions of motion (*P* < .05). In addition, when comparing the data of both types of disc prostheses directly, we found a little higher values of total ROM and segmental ROM at C5/6 with the CDR Type P prosthesis than with the CDR Type D prosthesis without statistical significance (Fig. 6A–C).

**Table 1:**
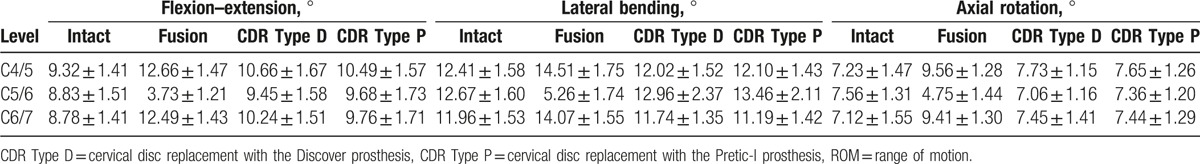
Segmental ROM in flexion–extension, lateral bending, and axial rotation in 4 different models.

### 3.2 Pressure analysis

**Figure 7:**
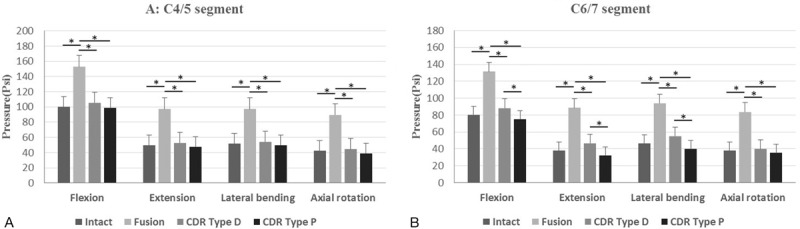
IDP at the adjacent segments. IDP at C4/5 (A) and C6/7 (B) in flexion, extension, lateral bending, and axial rotation in 4 different models: intact, fusion, CDR Type D, and CDR Type P. Statistically significant differences are denoted by ∗, with bars connecting the corresponding columns. CDR = cervical disc replacement, IDP = intradiscal pressure.

The differences in the IDP at C4/5 among the 3 models (intact, CDR Type D, and CDR Type P) were not statistically significant in all 3 directions of motion. However, the IDP at C4/5 after fusion was significant larger than that in the other 3 models in flexion, extension, lateral bending, and axial rotation (*P* < .05) (Fig. 7A). In addition, the differences in the IDP at C6/7 between the state of intact and CDR were also not statistically significant in all 3 directions of motion. Whereas the IDP at C6/7 after fusion was significant larger than that in the other 3 models in flexion, extension, lateral bending, and axial rotation (*P* < .05). Besides, when comparing both prostheses directly, we found that the IDP at C6/7 after CDR with Type P prosthesis was lower than that after CDR with Type D prosthesis in flexion, extension, and lateral bending with significant difference (*P* < .05), but not under axial rotation (Fig. 7B).

## 4 Discussion

As CDR become more and more popular in the operative management of cervical degenerative disc diseases, in vitro studies are very important to investigate the biomechanical behavior of the different prostheses. In our study, we tested the kinematics of the cadaveric cervical specimens under 4 different conditions (intact, simulated fusion, and CDR with 2 types of prostheses). Furthermore, the IDP at the adjacent segments was also analyzed.

As expected, total ROM after fusion decreased significantly in all 3 directions of motion, compared with the other 3 models: intact, CDR Type D, and CDR Type P (*P* < .05). However, when comparing the data of both types of disc prostheses directly, we found a little higher values of total ROM with the CDR Type P prosthesis than with the CDR Type D prosthesis, without statistical significance. In addition, the ROM at C5/6 after fusion was significant smaller than that in the other 3 models (*P* < .05). However, the ROM at adjacent segments after fusion was both significant larger than that in the other 3 models (*P* < .05). After CDR with Type P prosthesis and Type D prosthesis, the segmental and total ROM approximated to the values of the intact model without statistical significance, similar to previous studies.^[13–15]^ Based on the results above, CDR with Type P prosthesis was able to maintain the ROM at the target segment, and did not affect the ROM at the adjacent segments as well as total ROM. Moreover, compared with the Type D prosthesis, CDR with Type P prosthesis presented a little higher values of total ROM and segmental ROM at the target segment without statistical significance. This is mainly attributed to the design characteristic of Type P prosthesis with a mobile core, which has the theoretical advantage of providing normal kinematics over a fixed-core prosthesis.^[8]^

When discussing the IDP of adjacent segments, we have to state that we did not analyze the peak values but the mean data at the extreme position when the pure moment reached the maximum 2 Nm in flexion, extension, lateral bending, and axial rotation. Similar to the change trend of the ROM at the adjacent segments after fusion, the IDP was significantly larger at C4/5 and C6/7 in flexion, extension, lateral bending, and axial rotation, compared to the intact model and CDR with 2 types of prostheses. After arthroplasty, the mean values of the IDP at adjacent segments approximated to the values of the intact model, similar to previous studies.^[16,17]^ Nonetheless, when comparing both prostheses directly, there was no significant difference in the IDP at C4/5, meanwhile the IDP at C6/7 after CDR with Type P prosthesis was lower than that after CDR with Type D prosthesis in flexion, extension, and lateral bending with significant difference (*P* < .05), but not under axial rotation, perhaps with a lower risk of adjacent segment degeneration. Based on our understanding, we speculated that the differences in IDP between 2 prostheses should be attributed to the 3 following causes. First, the novel prosthesis was designed with an arcuate surface in attempt to increase the contact area between the prosthesis and cervical endplate, and to dissipate the axial load further.^[18]^ Second, the novel prosthesis was designed with a mobile core so as to have a disperse pressure at the prosthesis–bone interface, as the core of the prosthesis is able to translate, despite its insertion point slightly missing the center of intervertebral disc space.^[19]^ Again, the design features of the Discover prosthesis with a fixed core require precise device placement to replicate anatomical centers of rotation. When its location misses the center, inappropriate placement of a fixed-core prosthesis can theoretically increase the pressure at the prosthesis–bone interface, and even increase the risk of accelerated adjacent segment changes.^[20,21]^ In addition, facet loads were not measured in this study, as there has always been a controversy about the influence of CDR with a mobile- or fixed-core prosthesis on the facet loads. Some previous studies indicated that CDR with a fixed-core prosthesis has lower pressure on the facet joint than CDR with a mobile-core prosthesis.^[20,22]^ However, the opposite opinion was also reported that CDR with a mobile-core prosthesis reduced the pressure on the facet joint more than CDR with a fixed-core prosthesis.^[23]^

In this work, we performed the biomechanical evaluation of CDR with a novel prosthesis (Pretic-I). Moreover, we analyzed the differences between fixed-core (Discover, DePuy) and mobile-core (Pretic-I, Trauson) artificial disc prostheses. However, this study still has several limitations that should be acknowledged. First, only a limited number of samples was studied, leading to potential Type 2 (false negative) statistical errors. Second, the specimens were tested as intact model, fusion, CDR with Type D prosthesis, and CDR with Type P prosthesis in sequence, regardless of the effect of reduplicative surgical procedures on the accuracy of measuring outcomes. In addition, the ROM of cervical spine and IDP of adjacent segments were both measured and calculated at the extreme position when the pure moment reached the maximum 2 Nm. So, we are unable to know the changing process and actual peak values for cervical ROM and IDP of adjacent segments. Lastly, facet loads were not measured. Therefore, it was impossible to comprehensively evaluate the influence of CDR on the load distribution at the target segment and adjacent segments.

## 5 Conclusion

In summary, CDR with a novel prosthesis was effective to maintain the ROM at the target segment and did not affect the ROM and IDP at the adjacent segments. Moreover, CDR with a mobile-core prosthesis presented a little higher values of target segment and total ROM, but lower IDP at the inferior adjacent segment than CDR with a fixed-core prosthesis. However, due to the limitations of this study, further investigations with larger sample sizes are essential to verify the current findings. Moreover, facet loads should also be measured in the future studies.
